# Supplementary material for: Regulation of the Expression of nucS, a Key Component of the Mismatch Repair System in Mycobacteria
Source: Antibiotics (Basel). 2025 Oct 24;14(11):1065. doi: 10.3390/antibiotics14111065 (PMC12649562; doi:10.3390/antibiotics14111065)
Supplement: Supplementary file 1 [file antibiotics-14-01065-s001.zip › antibiotics-3919492-supplementary.pdf]

antibiotics

MDPI

Supplementary Materials

Supplementary Tables

Table S1. Vectors used in this study.

Plasmid	Characteristics	Reference or origin
**pSGV53**	Replicative plasmid in mycobacteria (*oriM*, pAL5000) and *E. coli* (*oriE*, pBR322). Antibiotic resistance marker for the bleomycin family, including zeocin (*bleo*^{R}). Carries the *egfp* gene under the control of the constitutive expression promoter P_{mpt64}.	Gola *et al.*, 2015 [26]
**pSGV53-P_{nucS}-73-*gfp***	Reporter vector containing a transcriptional fusion of 73 bp upstream of *nucS* followed by *gfp*. Plasmid backbone from pSGV53 (*oriM*, *oriE*, *bleo*^{R}).	This work
**pSGV53-P_{nucS}-408-*gfp***	Reporter vector with a transcriptional fusion of 408 bp upstream of *nucS* followed by *gfp*. Plasmid backbone from pSGV53 (*oriM*, *oriE*, *bleo*^{R}).	This work
**pSGV53-P_{nucS}-408Δ46-*gfp***	Reporter vector with a transcriptional fusion with the 408 bp region upstream of *nucS*, excluding the first 46 bp (the intergenic region), followed by *gfp*. Plasmid backbone from pSGV53 (*oriM*, *oriE*, *bleo*^{R}).	This work
**p2NIL**	Suicide plasmid used for genetic manipulation of mycobacteria. It contains a multiple cloning site and resistance markers for kanamycin (*kan*^{R}, *aph*) and ampicillin (*amp*^{R}, *bla*). High-copy-number origin of replication in *E. coli* (*oriE*, pBR322).	Parish and Stoker, 2000 [52]
**pGOAL19**	Suicide plasmid in mycobacteria carrying a «hyg P_{Ag85}-*lacZ*-P_{hsp60}-*sacB* PacI» cassette. Origin of replication from *E. coli* (*oriE*, pBR322), *amp*^{R}.	Parish and Stoker, 2000 [52]

Table S2. Compounds tested from the Biolog™ Phenotype MicroArrays™.

Compound	Biolog™ Plate
Amikacin	*PM11C MicroPlate*™
Chlortetracycline	*PM11C MicroPlate*™
Lincomycin	*PM11C MicroPlate*™
Amoxicillin	*PM11C MicroPlate*™
Cloxacillin	*PM11C MicroPlate*™
Lomefloxacin	*PM11C MicroPlate*™
Bleomycin	*PM11C MicroPlate*™
Colistin	*PM11C MicroPlate*™
Minocycline	*PM11C MicroPlate*™
Capreomycin	*PM11C MicroPlate*™
Demeclocycline	*PM11C MicroPlate*™
Nafcillin	*PM11C MicroPlate*™
Cefazolin	*PM11C MicroPlate*™
Enoxacin	*PM11C MicroPlate*™
Nalidixic acid	*PM11C MicroPlate*™
Chloramphenicol	*PM11C MicroPlate*™
Erythromycin	*PM11C MicroPlate*™
Neomycin	*PM11C MicroPlate*™
Ceftriaxone	*PM11C MicroPlate*™
Gentamicin	*PM11C MicroPlate*™
Potassium tellurite	*PM11C MicroPlate*™
Cephalothin	*PM11C MicroPlate*™
Kanamycin	*PM11C MicroPlate*™
Ofloxacin	*PM11C MicroPlate*™
Penicillin G	*PM12B MicroPlate*™
Tetracycline	*PM12B MicroPlate*™
Carbenicillin	*PM12B MicroPlate*™
Oxacillin	*PM12B MicroPlate*™
Penimepicycline	*PM12B MicroPlate*™
Polymyxin B	*PM12B MicroPlate*™
Paromomycin	*PM12B MicroPlate*™
Vancomycin	*PM12B MicroPlate*™
D,L-Serine hydroxamate	*PM12B MicroPlate*™
Sisomicin	*PM12B MicroPlate*™
Sulfamethazine	*PM12B MicroPlate*™

Novobiocin	*PM12B MicroPlate™*
2,4-Diamino-6,7-diisopropylpteridine	*PM12B MicroPlate™*
Sulfadiazine	*PM12B MicroPlate™*
Benzethonium chloride	*PM12B MicroPlate™*
Tobramycin	*PM12B MicroPlate™*
Sulfathiazole	*PM12B MicroPlate™*
5-Fluoroorotic acid	*PM12B MicroPlate™*
Spectinomycin	*PM12B MicroPlate™*
Sulfamethoxazole	*PM12B MicroPlate™*
L-Aspartic-β-hydroxamate	*PM12B MicroPlate™*
Spiramycin	*PM12B MicroPlate™*
Rifampicin	*PM12B MicroPlate™*
Dodecyltrimethyl ammonium bromide	*PM12B MicroPlate™*
Ampicillin	*PM13B MicroPlate™*
Dequalinium chloride	*PM13B MicroPlate™*
Nickel chloride	*PM13B MicroPlate™*
Azlocillin	*PM13B MicroPlate™*
2, 2'-Dipyridyl	*PM13B MicroPlate™*
Oxolinic acid	*PM13B MicroPlate™*
6-Mercaptopurine	*PM13B MicroPlate™*
Doxycycline	*PM13B MicroPlate™*
Potassium chromate	*PM13B MicroPlate™*
Cefuroxime	*PM13B MicroPlate™*
5-Fluorouracil	*PM13B MicroPlate™*
Rolitetracycline	*PM13B MicroPlate™*
Cytosine-1-beta-D-arabinofuranoside	*PM13B MicroPlate™*
Geneticin (G418)	*PM13B MicroPlate™*
Ruthenium red	*PM13B MicroPlate™*
Cesium chloride	*PM13B MicroPlate™*
Glycine	*PM13B MicroPlate™*
Thallium (I) acetate	*PM13B MicroPlate™*
Cobalt chloride	*PM13B MicroPlate™*
Manganese chloride	*PM13B MicroPlate™*
Trifluoperazine	*PM13B MicroPlate™*
Cupric chloride	*PM13B MicroPlate™*
Moxalactam	*PM13B MicroPlate™*
Tylosin	*PM13B MicroPlate™*
Acriflavine	*PM14A MicroPlate™*

Furaltadone	*PM14A MicroPlate*™
Sanguinarine	*PM14A MicroPlate*™
9-Aminoacridine	*PM14A MicroPlate*™
Fusaric acid	*PM14A MicroPlate*™
Sodium arsenate	*PM14A MicroPlate*™
Boric Acid	*PM14A MicroPlate*™
1-Hydroxypyridine -2-thione	*PM14A MicroPlate*™
Sodium cyanate	*PM14A MicroPlate*™
Cadmium chloride	*PM14A MicroPlate*™
Iodoacetate	*PM14A MicroPlate*™
Sodium dichromate	*PM14A MicroPlate*™
Cefoxitin	*PM14A MicroPlate*™
Nitrofurantoin	*PM14A MicroPlate*™
Sodium metaborate	*PM14A MicroPlate*™
Chloramphenicol	*PM14A MicroPlate*™
Piperacillin	*PM14A MicroPlate*™
Sodium metavanadate	*PM14A MicroPlate*™
Chelerythrine	*PM14A MicroPlate*™
Carbenicillin	*PM14A MicroPlate*™
Sodium nitrite	*PM14A MicroPlate*™
EGTA	*PM14A MicroPlate*™
Promethazine	*PM14A MicroPlate*™
Sodium orthovanadate	*PM14A MicroPlate*™
Procaine	*PM15B MicroPlate*™
Guanidine hydrochloride	*PM15B MicroPlate*™
Cefmetazole	*PM15B MicroPlate*™
D-Cycloserine	*PM15B MicroPlate*™
EDTA	*PM15B MicroPlate*™
5,7-Dichloro- 8-hydroxyquinaldine	*PM15B MicroPlate*™
5,7-Dichloro-8-hydroxyquinoline	*PM15B MicroPlate*™
Fusidic acid	*PM15B MicroPlate*™
1,10-Phenanthroline	*PM15B MicroPlate*™
Phleomycin	*PM15B MicroPlate*™
Domiphen bromide	*PM15B MicroPlate*™
Nordihydroguaia retic acid	*PM15B MicroPlate*™
Alexidine	*PM15B MicroPlate*™
5-Nitro-2-furaldehyde semicarbazone	*PM15B MicroPlate*™
Methyl viologen	*PM15B MicroPlate*™

3, 4-Dimethoxybenzyl alcohol	*PM15B MicroPlate™*
Oleandomycin	*PM15B MicroPlate™*
Puromycin	*PM15B MicroPlate™*
CCCP	*PM15B MicroPlate™*
Sodium azide	*PM15B MicroPlate™*
Menadione	*PM15B MicroPlate™*
2-Nitroimidazole	*PM15B MicroPlate™*
Hydroxyurea	*PM15B MicroPlate™*
Zinc chloride	*PM15B MicroPlate™*
Cefotaxime	*PM16A MicroPlate™*
Phosphomycin	*PM16A MicroPlate™*
5-Chloro-7-iodo-8-hydroxyquinoline	*PM16A MicroPlate™*
Norfloxacin	*PM16A MicroPlate™*
Sulfanilamide	*PM16A MicroPlate™*
Trimethoprim	*PM16A MicroPlate™*
Dichlofluanid	*PM16A MicroPlate™*
Protamine sulfate	*PM16A MicroPlate™*
Cetylpyridinium chloride	*PM16A MicroPlate™*
1-Chloro -2,4-dinitrobenzene	*PM16A MicroPlate™*
Diamide	*PM16A MicroPlate™*
Cinoxacin	*PM16A MicroPlate™*
Streptomycin	*PM16A MicroPlate™*
5-Azacytidine	*PM16A MicroPlate™*
Rifamycin SV	*PM16A MicroPlate™*
Potassium tellurite	*PM16A MicroPlate™*
Sodium selenite	*PM16A MicroPlate™*
Aluminum sulfate	*PM16A MicroPlate™*
Chromium chloride	*PM16A MicroPlate™*
Ferric chloride	*PM16A MicroPlate™*
L-Glutamic-ghydroxamate	*PM16A MicroPlate™*
Glycine hydroxamate	*PM16A MicroPlate™*
Chloroxylenol	*PM16A MicroPlate™*
Sorbic acid	*PM16A MicroPlate™*
D-Serine	*PM17A MicroPlate™*
β-Chloro-L-alanine hydrochloride	*PM17A MicroPlate™*
Thiosalicylic acid	*PM17A MicroPlate™*
Sodium salicylate	*PM17A MicroPlate™*
Hygromycin B	*PM17A MicroPlate™*

Ethionamide	*PM17A MicroPlate*™
4-Aminopyridine	*PM17A MicroPlate*™
Sulfachloropyridazine	*PM17A MicroPlate*™
Sulfamonomethoxine	*PM17A MicroPlate*™
Oxycarboxin	*PM17A MicroPlate*™
3-Amino-1,2,4-triazole	*PM17A MicroPlate*™
Chlorpromazine	*PM17A MicroPlate*™
Niaproof	*PM17A MicroPlate*™
Compound 48/80	*PM17A MicroPlate*™
Sodium tungstate	*PM17A MicroPlate*™
Lithium chloride	*PM17A MicroPlate*™
DL-Methionine hydroxamate	*PM17A MicroPlate*™
Tannic acid	*PM17A MicroPlate*™
Chlorambucil	*PM17A MicroPlate*™
Cefamandole nafate	*PM17A MicroPlate*™
Cefoperazone	*PM17A MicroPlate*™
Cefsulodin	*PM17A MicroPlate*™
Caffeine	*PM17A MicroPlate*™
Phenylarsine oxide	*PM17A MicroPlate*™
Ketoprofen	*PM18C MicroPlate*™
Sodium pyrophosphate decahydrate	*PM18C MicroPlate*™
Thiamphenicol	*PM18C MicroPlate*™
Trifluorothymidin	*PM18C MicroPlate*™
Pipemidic Acid	*PM18C MicroPlate*™
Azathioprine	*PM18C MicroPlate*™
Poly-L-lysine	*PM18C MicroPlate*™
Sulfisoxazole	*PM18C MicroPlate*™
Pentachlorophenol	*PM18C MicroPlate*™
Sodium m-arsenite	*PM18C MicroPlate*™
Sodium bromate	*PM18C MicroPlate*™
Lidocaine	*PM18C MicroPlate*™
Sodium metasilicate	*PM18C MicroPlate*™
Sodium m-periodate	*PM18C MicroPlate*™
Antimony (III) chloride	*PM18C MicroPlate*™
Semicarbazide	*PM18C MicroPlate*™
Tinidazole	*PM18C MicroPlate*™
Aztreonam	*PM18C MicroPlate*™
Triclosan	*PM18C MicroPlate*™

3,5-Diamino-1,2,4-triazole (Guanazole)	*PM18C MicroPlate*™
Myricetin	*PM18C MicroPlate*™
5-fluoro-5′- deoxyuridine	*PM18C MicroPlate*™
2-Phenylphenol	*PM18C MicroPlate*™
Plumbagin	*PM18C MicroPlate*™
Josamycin	*PM19 MicroPlate*™
Gallic acid	*PM19 MicroPlate*™
Coumarin	*PM19 MicroPlate*™
Methyltrioctylammonium chloride	*PM19 MicroPlate*™
Harmane	*PM19 MicroPlate*™
2,4-Dintrophenol	*PM19 MicroPlate*™
Chlorhexidine	*PM19 MicroPlate*™
Umbelliferone	*PM19 MicroPlate*™
Cinnamic acid	*PM19 MicroPlate*™
Disulphiram	*PM19 MicroPlate*™
Iodonitro Tetrazolium Violet	*PM19 MicroPlate*™
Phenyl- methylsulfonylfluoride (PMSF)	*PM19 MicroPlate*™
FCCP	*PM19 MicroPlate*™
D,L-Thioctic Acid	*PM19 MicroPlate*™
Lawsone	*PM19 MicroPlate*™
Phenethicillin	*PM19 MicroPlate*™
Blasticidin S	*PM19 MicroPlate*™
Sodium caprylate	*PM19 MicroPlate*™
Lauryl sulfobetaine	*PM19 MicroPlate*™
Dihydrostreptomycin	*PM19 MicroPlate*™
Hydroxylamine	*PM19 MicroPlate*™
Hexammine cobalt (III) chloride	*PM19 MicroPlate*™
Thioglycerol	*PM19 MicroPlate*™
Polymyxin B	*PM19 MicroPlate*™
Amitriptyline	*PM20B MicroPlate*™
Apramycin	*PM20B MicroPlate*™
Benserazide	*PM20B MicroPlate*™
Orphenadrine	*PM20B MicroPlate*™
D,L-Propranolol	*PM20B MicroPlate*™
Tetrazolium violet	*PM20B MicroPlate*™
Thioridazine	*PM20B MicroPlate*™
Atropine	*PM20B MicroPlate*™
Ornidazole	*PM20B MicroPlate*™

Proflavine	*PM20B MicroPlate™*
Ciprofloxacin	*PM20B MicroPlate™*
18-Crown-6-ether	*PM20B MicroPlate™*
Crystal violet	*PM20B MicroPlate™*
Dodine	*PM20B MicroPlate™*
Hexachlorophene	*PM20B MicroPlate™*
4-Hydroxycoumarin	*PM20B MicroPlate™*
Oxytetracycline	*PM20B MicroPlate™*
Pridinol	*PM20B MicroPlate™*
Captan	*PM20B MicroPlate™*
3,5-Dinitrobenzene	*PM20B MicroPlate™*
8-Hydroxyquinoline	*PM20B MicroPlate™*
Patulin	*PM20B MicroPlate™*
Tolylfluanid	*PM20B MicroPlate™*
Troleandomycin	*PM20B MicroPlate™*

Table S3. Compounds tested in the disk diffusion assay.

Compound	Putative inducer/repressor	Solvent	Concentration of stock solution
Chlortetracycline hydrochloride	Inducer	Water	8 mg/ml
Tetracycline hydrochloride	Inducer	Water	5 mg/ml
Cytosine-1-β-D-arabinofuranoside	Inducer	Water	190 mg/ml
5,7-dichloro-8-hydroxyquinaldine	Inducer	Acetone	50 mg/ml
Sulfanilamide	Inducer	DMSO	50 mg/ml
Thiosalicylic acid	Inducer	DMSO	150 mg/ml
Sodium tungstate dihydrate	Inducer	Water	300 mg/ml
Sodium pyrophosphate decahydrate	Inducer	Water	50 mg/ml
Umbelliferone	Inducer	Ethanol	10 mg/ml
4-hydroxycoumarin	Inducer	DMSO	150 mg/ml
Sulfadiazine	Inducer	DMSO	2 mg/ml
5,7-dichloro-8-hydroxyquinoline	Inducer	Acetone	35 mg/ml
8-hydroxyquinoline	Inducer	Ethanol	0.9 mg/ml
Cupric chloride	Repressor	Water	20 mg/ml
Sodium selenite	Repressor	Water	170 mg/ml
Tannic acid	Repressor	Water	200 mg/ml
Chlorambucil	Repressor	Ethanol	100 mg/ml
2-phenylphenol	Repressor	Ethanol	150 mg/ml
Gallic acid monohydrate	Repressor	Ethanol	150 mg/ml
D,L-Thioctic acid	Repressor	Ethanol	100 mg/ml
Lawsone	Repressor	DMSO	10 mg/ml
1-chloro-2,4-dinitrobenzene	Repressor	Ethanol	1 mg/ml

Table S4. Primers used in this study.

**Name**[a]	**Sequence (5′ → 3′)**[b]
**5′-RACE**	
GSP-RT (r)	TCCTGTTCGGTGACCCAG
Q_T	CCAGTGAGCAGAGTGACGAGGACTCGAGCTCAGCTTTTTTTTTTTTTTTTT
Q_O	CCAGTGAGCAGAGTGACG
Q_I	GAGGACTCGAGCTCAAGC
GSP1 (r)	TAACTGCAGCAGCACGGCGGGCTCATC
GSP2 (r)	CACCTGCAGCTCATCCAGTTCAACGGC
**Construction of GFP reporter plasmids**	
73up_nucS_NotI_F (f)	TAC<u>GCGGCCGC</u>CCGCGCCAGCGAATTGTCGGCG
408up_nucS_NotI_F (f)	TAA<u>GCGGCCGC</u>ACGCTGGGGGACGGGAAGAGC
nucS_up_NdeI_R (r)	TAA<u>CATATG</u>ATCCACCTTAAAGGCACCGCCG
msmeg4924startR (r)	ATAGCC<u>CATATG</u>AACGCCGACAATTCGCTG
ble_fw_1 (f) (seq)	CAGAATCGGTGGTTGTGGTGATG
gfp_rev_seq_2 (r) (seq)	CTTCAGGGTCAGCTTGCCGTAGGTG
**qPCR**	
sigA_Msm_qPCR_F (f)	GTGTGGGACGAGGAAGAGTC
sigA_Msm_qPCR_R (r)	ACCTCTTCTTCGGCGTTGAG
nucS_Msm_qPCR_F_2 (f)	TCGAGCATGATTCGCACCACG
nucS_Msm_qPCR_R_2 (r)	TCGGATACTCGCGGCGCAC
lpqM_TB_qPCR_F (f)	GATTCCAACGACCCAAGTAGTC
lpqM_TB_qPCR_R (r)	CGATCCCAGGCAATCAAGTT
nucS_TB_qPCR_F (f)	ACGGATCGGTCAGCGTACAT
nucS_TB_qPCR_R (r)	CACGGCGGACTCATCCA
**Construction of Δ*sigB* mutant**	
sigB5_PstIF (f)	TAA<u>CTGCAG</u>TGCGCAAGTGGATCGAGACCAG
sigB5_HindIIIR (r)	TCA<u>AAGCTT</u>GTCAACGCGGCTTGTGGTGGC
sigB3_HindIIIF (f)	GAT<u>AAGCTT</u>CGCTCCTACGCGAGCTGATGC
sigB3_BamHIR (r)	TAA<u>GGATCC</u>TCTCGGCGTGCAGTTACCGCCG
sigB5_end_F (f)	GCTGATGTCCGATCTCCACCG
sigB3_start_R (r)	ACAAGATCGTTCATGCGCGCC
sigB.intF (f)	GTCCACCTCGTCGAGCAGGTC
sigB.intR (r)	TGCAGAAGCTCCGAGATGACG

Supplementary Figures

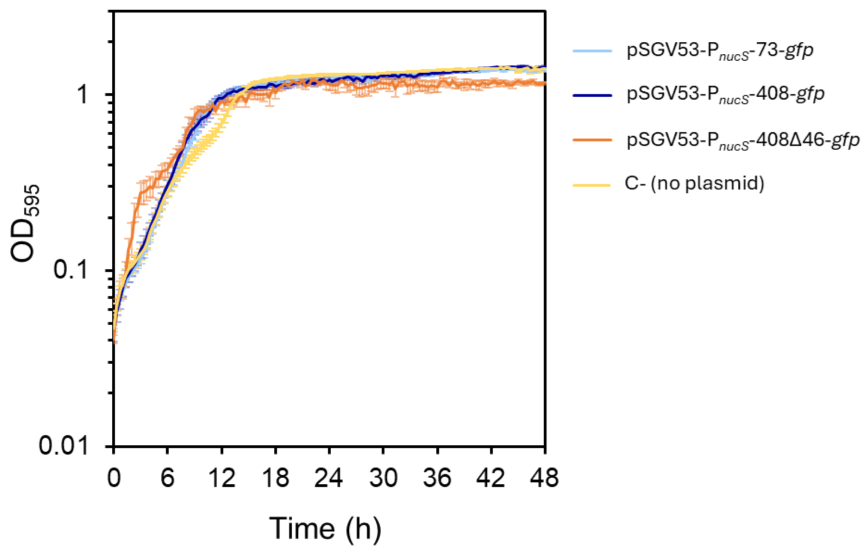

Figure S1. Growth curves of the reporter strains in the microplate reader. OD_{595} values were recorded during growth using an Infinite® 200 spectrofluorometer (TECAN) (see *Materials and Methods*). Data represent the mean ± standard error (SE) of eight biological replicates (n=8).

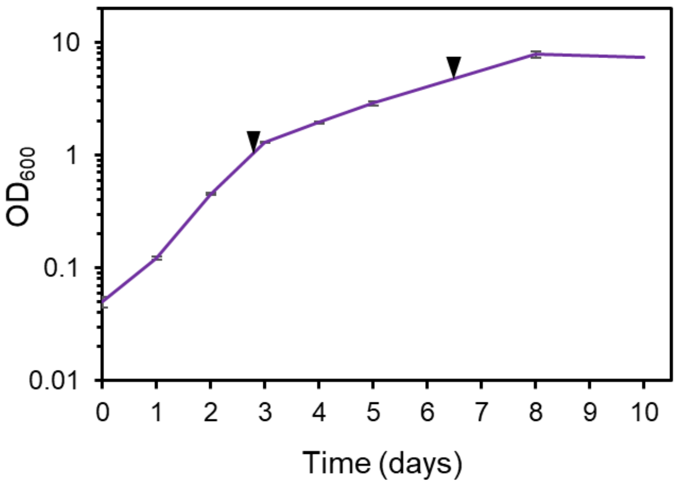

Figure S2. Growth curve of *M. tuberculosis* H37Rv. Cultures were grown in 7H9 broth supplemented with 0.5% glycerol, 0.05% Tween 80 and 10% OADC, and incubated in roller bottles at 37°C. Black triangles indicate the points at which samples were collected for RNA extraction. Data represent the mean OD_{600} ± standard error (SE) of four biological replicates.

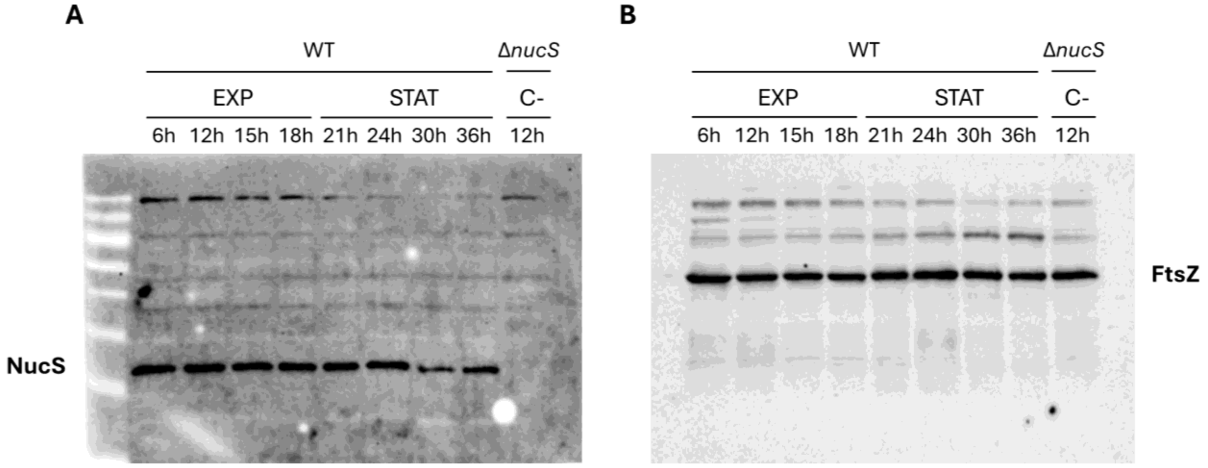

Figure S3. Full membrane images from a representative Western blot experiment. **(A)** Detection of NucS in *M. smegmatis* protein extracts collected at the indicated time points. The membrane was incubated with a mouse anti-NucS antibody followed by a goat anti-mouse IgG-HRP secondary antibody (see *Materials and Methods* section). In the left lane, the Color Prestained Protein Standard, Broad Range (10-250 kDa) (New England Biolabs) is shown. **(B)** Detection of FtsZ bands in the same samples, used as a loading control. After NucS detection, the membrane was treated with sodium azide to inactivate residual peroxidase activity and then incubated with a rabbit anti-FtsZ antibody followed by HRP-Protein A (see *Materials and Methods* section). No NucS signal was observed when HRP-Protein A was used. A Δ*nucS* extract collected at 12 h was included as a negative control. EXP: exponential phase; STAT: stationary phase. Signal detection was performed using ECL reagents and visualized with a ChemiDoc™ Touch Imaging System (Bio-Rad).

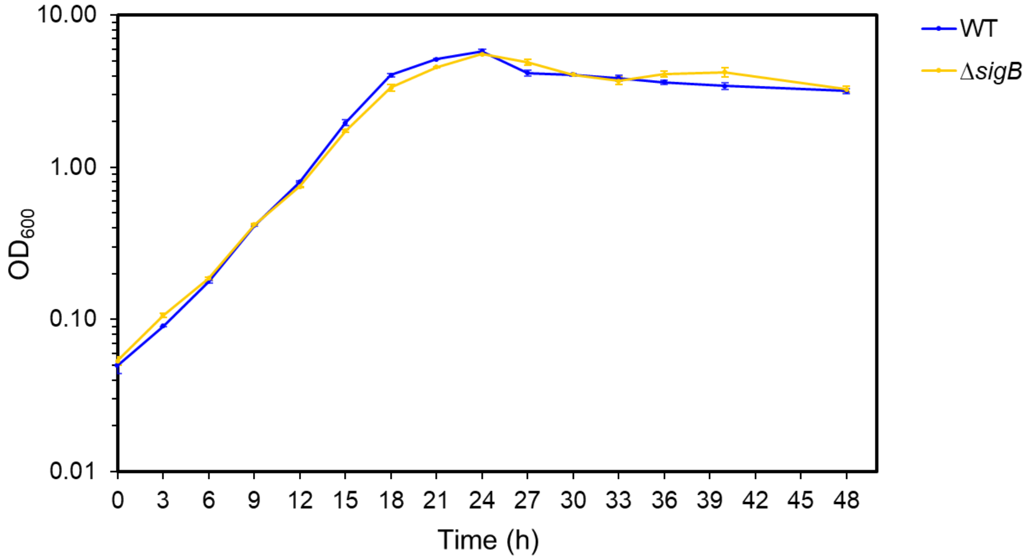

Figure S4. Growth curves of WT and Δ*sigB* strains of *M. smegmatis* mc[2] 155. Growth of WT (blue) and Δ*sigB* (yellow) strains over 48 h. Both strains exhibited similar growth rates. Cultures were initiated at OD_{600} = 0.05 and incubated at 37ºC with orbital shaking (250 rpm). Error bars represent the standard error of three biological replicates.

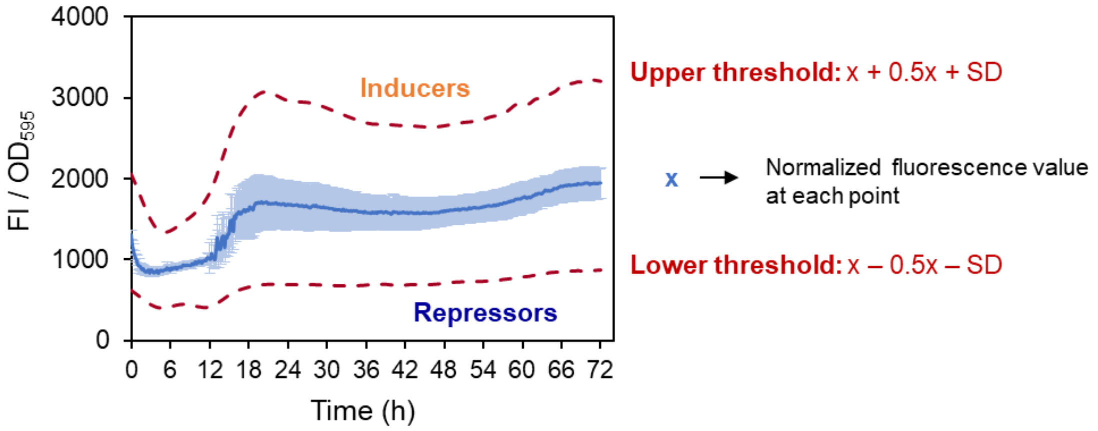

Figure S5. Selection of thresholds for filtering candidate compounds regulating *nucS* expression. The blue line represents the mean normalized fluorescence relative to OD (FI/OD_{595}) over 72 hours of growth. Light blue error bars indicate the standard deviation (SD) (n=7). Dashed red lines show the upper and lower threshold values.

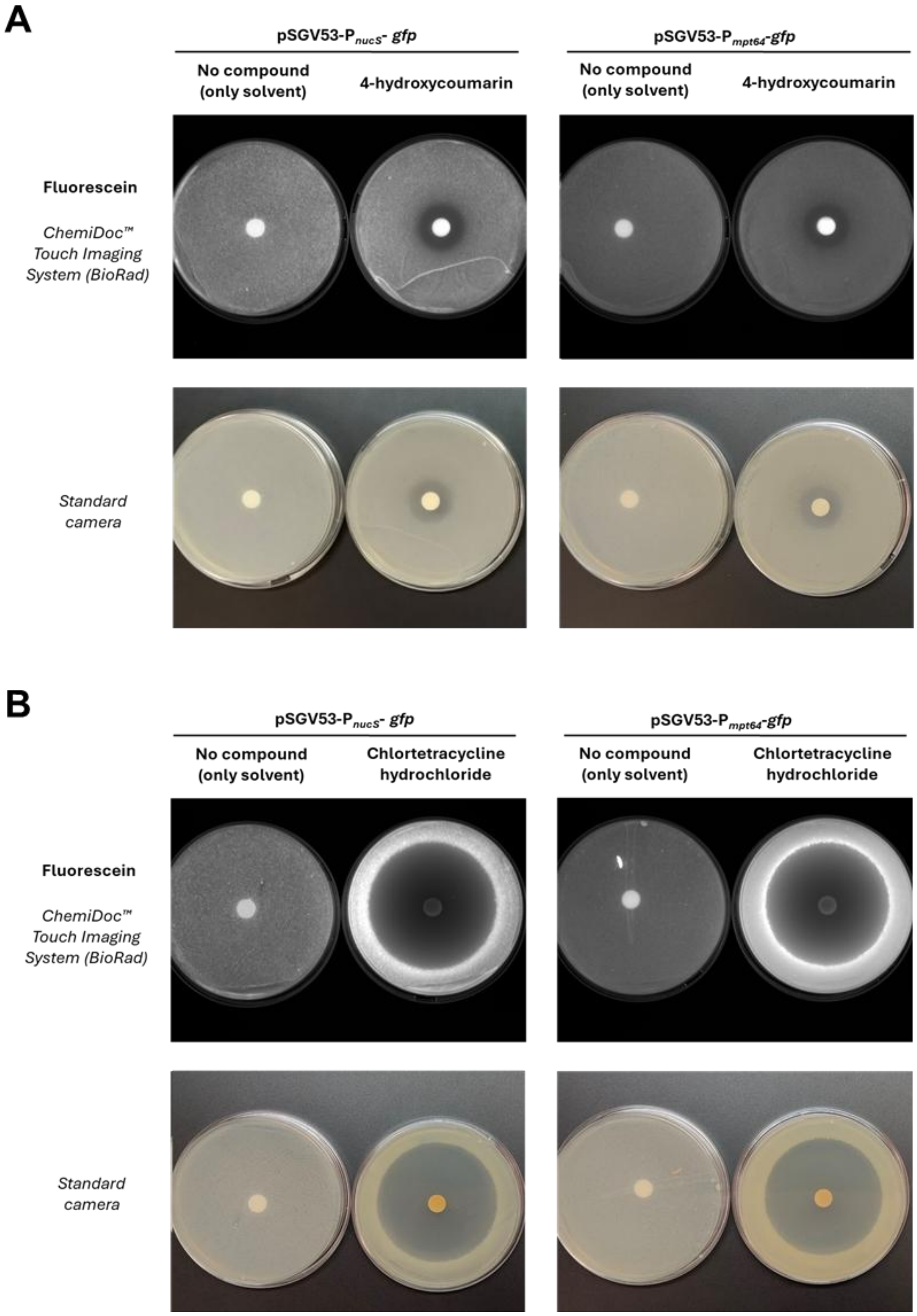

Figure S6. Example of results from disk diffusion assays for discarded compounds. Top panels in both A and B (fluorescence): GFP fluorescence of the *M. smegmatis nucS*::*gfp* reporter strain (left panel) and a constitutive GFP-expressing control strain (right panel), each exposed to the test compound (right disk) and solvent control (left disk). Bottom panels in both A and B: Corresponding photographs of the same plates showing cell mass distribution. **(A)** Disk diffusion assay with 4-hydroxycoumarin, a discarded candidate compound with no detectable effects on *nucS* expression. No visible increase or decrease in the fluorescence around the inhibition halo was observed in either strain. **(B)** Disk diffusion assay with chlortetracycline, a discarded candidate compound showing nonspecific effects on *nucS* expression. Increased fluorescence around the inhibition halo is visible in both strains, indicating a nonspecific effect on protein expression.
